# The Japanese Catheter Ablation Registry (J‐AB): A prospective nationwide multicenter registry in Japan. Annual report in 2018

**DOI:** 10.1002/joa3.12445

**Published:** 2020-10-16

**Authors:** Kengo Kusano, Teiichi Yamane, Koichi Inoue, Misa Takegami, Yoko M. Nakao, Yoshihiro Miyamoto, Morio Shoda, Akihiko Nogami, J‐AB registry investigators

**Affiliations:** ^1^ Department of Cardiovascular Medicine National Cerebral and Cardiovascular Center Osaka Japan; ^2^ Division of Cardiology Department of Internal Medicine The Jikei University School of Medicine Tokyo Japan; ^3^ Cardiovascular Center Sakurabashi Watanabe Hospital Osaka Japan; ^4^ Department of Preventive Medicine and Epidemiologic Informatics National Cerebral and Cardiovascular Center Suita Japan; ^5^ Center for Cerebral and Cardiovascular Disease Information Open Innovation Center National Cerebral and Cardiovascular Center Suita Japan; ^6^ Clinical Research Division of Heart Rhythm Management Department of Cardiology Tokyo Women's Medical University Tokyo Japan; ^7^ Department of Cardiology Faculty of Medicine University of Tsukuba Tsukuba Japan

**Keywords:** catheter ablation, complication, J‐AB, REDCap, registry

## Abstract

**Background:**

To analyze the type of ablation procedure, acute outcomes, and complications related to catheter ablation in Japan during the year of 2018.

**Method:**

The Japanese Catheter Ablation (J‐AB) registry is a voluntary, nationwide, multicenter, prospective, observational registry, performed by the Japanese Heart Rhythm Society (JHRS) in collaboration with the National Cerebral and Cardiovascular Center using a Research Electronic Data Capture system. The procedural outcome and complications during hospitalizations were collected.

**Result:**

A total of 55 525 procedures (mean age of 64.5 years and 66.5% male) from 369 hospitals were collected. The total number of target arrhythmias was 61 610 including atrial fibrillation (AF, 65.6%), atrial flutter (AFL) or atrial tachycardia (16.7%), atrioventricular nodal reentrant tachycardia (7.4%), atrioventricular reentrant tachycardia (3.5%), premature ventricular contractions (4.1%), and ventricular tachycardia (VT, 2.0%). Over a 90% acute success rate was observed among all arrhythmias except for VT due to structural heart disease, and notably, an over 99% success rate was achieved for pulmonary vein isolation of AF and inferior vena cava‐tricuspid valve isthmus block for isthmus‐dependent AFL. Acute complications during hospitalization were observed in 1558 patients (2.8%), including major bleeding (Bleeding Academic Research Consortium: BARC criteria ≥2) in 1.1%, cerebral or systemic embolisms in 0.2%, and death in 0.1%. Acute complications were more often observed with AF ablation (*P* < .001), especially the first AF ablation session and with structural heart disease (*P* < .001).

**Conclusion:**

The J‐AB registry provided real‐world data regarding the acute outcomes and complications of ablation for the various types of arrhythmias in Japan.

## Graphical abstract

J‐AB is a prospective nationwide multicenter registry designed to collect clinical variables and sort‐term outcome data, aiming to register all catheter ablation cases performed in Japan. We successfully collected 55 525 procedures including 61 610 target arrhythmias during the year of 2018 and analyzed the acute success and complications during the hospitalization.

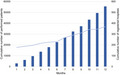

## 1 INTRODUCTION

Catheter ablation has become an established therapy for the management of various cardiac arrhythmias. It has been reported that catheter ablation is performed in up to 300‐600 patients per one million of the population annually in European countries^1^, ^2^ and the total number in Japan has exceeded 70 000 per year and is still increasing.^3^ However, little is known about the details of the ablation procedures including the type of target arrhythmias, outcomes, and acute complications related to the ablation procedure.

The Japanese Catheter Ablation (J‐AB) registry is a nationwide, multicenter, prospective, observational registry, performed by the Japanese Heart Rhythm Society (JHRS) in collaboration with the National Cerebral and Cardiovascular Center using a Research Electronic Data Capture (REDCap) system. This study was a nationwide registry and started in August 2017, since then, the number of participating medical instructions has increased from 125 at the end of 2017 to over 300 at the end of 2018. Now, we report the annual report of the J‐AB results during the year of 2018.

## 2 METHODS

The data from the J‐AB registry during the year of 2018 was used in this study. This nationwide registry was a voluntary one and started in August 2017. The method of this registry has been previously reported elsewhere.^4^ In brief, the study consisted of patients who were treated with any type of catheter ablation for any type of arrhythmia. Patients who received surgical management for arrhythmias were excluded from this registry. The data on the patient background (age, sex, and underlying heart diseases), target arrhythmias, outcome of the procedure (success rate of pulmonary vein isolation of atrial fibrillation [AF], inferior vena cava‐tricuspid [IVC‐TV] isthmus block for isthmus‐dependent atrial flutter [AFL], uncommon AFL or macro reentrant atrial tachycardia [AT], focal AT, slow pathway ablation of atrioventricular nodal reentrant tachycardia [AVNRT], accessory pathway ablation of atrioventricular reentrant tachycardia [AVRT], premature ventricular contractions [PVC], idiopathic ventricular tachycardia [VT], and ventricular tachycardia due to structural heart disease [ischemic or nonischemic cardiomyopathy or congenital heart disease]), and acute complications including major bleeding, cardiac tamponade, cerebral and systemic embolisms, phrenic nerve paralysis, esophageal injury, pericarditis, sick sinus syndrome, atrioventricular block, and in‐hospital death (cardiac, noncardiac, and unknown) during hospitalization were collected. Acute success was defined as complete success (no AT/VT inducible). Partial success was defined when the frequency or inducibility markedly decreased in cases in which the morphology of the clinical AT/VT was uncertain or the main targeted clinical AT/VT for treatment disappeared but some other ATs/VTs remained in cases with multiple AT/VT morphologies.

This study received the approval from the Institutional Review Board (IRB) of the National Cerebral and Cardiovascular Center (M28‐114‐7, approved at Dec 21, 2016), Japan, along with the IRBs of all participating hospitals. All participants were provided informed consent either by a written paper or by an opt out fashion and could withdraw their consent at any time. This study was also registered in the UMIN Clinical Trial Registry (UMIN 000028288) and ClinicalTrials.gov (NCT03729232).

### 2.1 Statistical analysis

The continuous variables were expressed as the means ± standard deviation (SD). Categorical data are reported with frequencies. Statistical tests of the differences were performed applying the Chi‐square test or Fisher's exact test as appropriate between patients with AF and non‐AF ablation, between the first and second or greater sessions for a different AF type, or between patients with and without structural heart disease. A *P* value < .05 was considered statistically significant, and all tests were carried out as two‐sided. Statistical analyses were carried out using STATA software (Stata Statistical Software: version 15, College Station, TX, USA).

## 3 RESULTS

**FIGURE 1:**
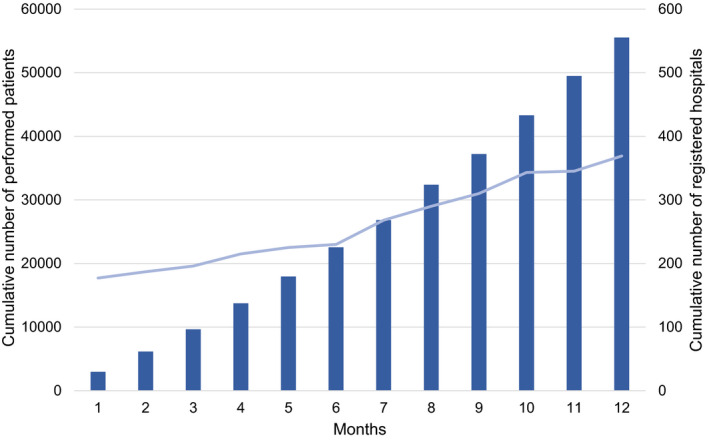
Cumulative number of hospitals participating in this registry (blue line) and the registered patients (blue bars) since January 2018

Figure 1 demonstrates the cumulative numbers of new registered patients and participating hospitals during the year of 2018. The number of patients and participating hospitals smoothly increased, and finally 55 525 procedures from 369 hospitals (mean 310 ± 223, median 249 procedures: IQR 144‐459/hospital), including 61 610 target arrhythmias, were registered during the year of 2018.

### 3.1 Patient background and target arrhythmias

**FIGURE 2:**
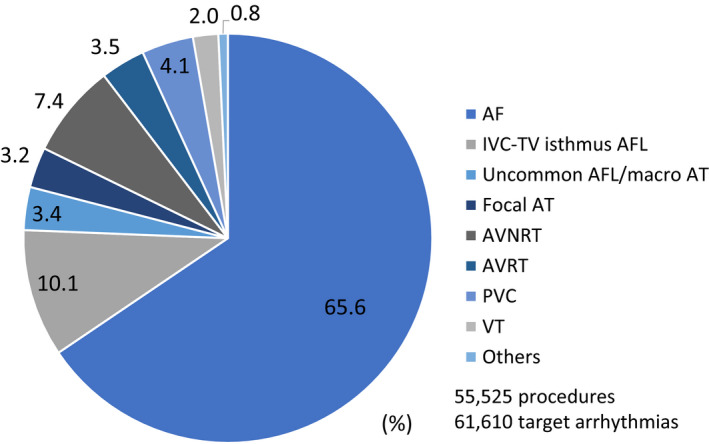
Distribution of the target arrhythmias in the J‐AB registry 2018. Note that around two‐thirds of the target arrhythmias were atrial fibrillation

The mean age was 64.5 ± 13.4 years, and 66.5% were male. Underlying heart diseases were observed in 10 577 patients (19.1%), including ischemic heart disease (IHD) in 6.1%, nonischemic cardiomyopathy in 5.4%, valvular disease in 3.2%, and congenital heart disease (CHD) in 1.3% (Table 1). The type of targeted arrhythmias were AF in 40 398 cases (65.6%) among all ablation procedures, IVC‐TV isthmus‐dependent AFL among 6202 cases (10.1%), uncommon AFL or macro reentrant AT among 2063 cases (3.4%), focal AT among 1982 cases (3.2%), AVNRT among 4557 cases (7.4%), AVRT among 2145 cases (3.5%), PVC among 2552 cases (4.1%), and VT among 1251 cases (2.0%) (Table 1 and Figure 2).

**TABLE 1:** Patient characteristics

	All procedures	All target arrhythmias	Mean ± standard deviation or n (%)						
			Atrial fibrillation (AF)^a^			Atrial flutter (AFL)/atrial tachycardia (AT)			
			All AF	Paroxysmal AF (PAF)	non‐PAF	All AFL/AT	IVC‐TV Isthmus dependent AFL	Uncommon AFL/macro AT	Focal AT
n	55 525	61 610	40 398	24 537	15 765	9492	6202	2063	1982
Age, y	64.5 ± 13.4	64.7 ± 13.3	66.4 ± 10.5	66.6 ± 10.8	66.1 ± 10.0	67.0 ± 13.1	67.7 ± 12.1	68.0 ± 12.1	64.0 ± 16.3
Male gender	36 894 (66.5)	41 067 (66.7)	28 247 (69.9)	16 197 (66.0)	11 980 (76.0)	6524 (68.7)	4765 (76.8)	1212 (58.8)	990 (50.0)
Heart diseases	10 577 (19.1)	12 187 (19.8)	7243 (17.9)	3858 (15.7)	3374 (21.4)	2838 (29.9)	1792 (28.9)	941 (45.6)	406 (23.0)
IHD	3391 (6.1)	3850 (6.3)	2331 (5.8)	1382 (5.6)	943 (6.0)	808 (8.5)	595 (9.6)	184 (8.9)	97 (4.9)
Cardiomyopathy	3015 (5.4)	3347 (5.6)	2062 (5.1)	892 (3.6)	1170 (7.4)	647 (6.8)	412 (6.6)	191 (9.3)	105 (5.3)
Valve disease	1748 (3.2)	2126 (3.5)	992 (2.5)	480 (2.0)	509 (3.2)	832 (8.8)	427 (6.9)	424 (20.6)	129 (6.5)
CHD	738 (1.3)	922 (1.5)	353 (0.9)	216 (0.9)	137 (0.9)	375 (4.0)	224 (3.6)	153 (7.4)	82 (4.1)

	Mean ± standard deviation or n (%)						
	Atrioventricular nodal reentrant tachycardia	Atrioventricular reentrant tachycardia	Premature ventricular contraction	Ventricular tachycardia (VT)			
				Idiopathic VT	VT due to ischemic cardiomyopathy	VT due to nonischemic cardiomyopathy	VT due to congenital heart disease
n	4557	2145	2552	594	316	326	15
Age, y	58.2 ± 17.1	47.8 ± 20.2	56.7 ± 16.5	53.3 ± 20.0	68.2 ± 10.7	63.4 ± 13.2	45.2 ± 18.1
Male gender	1936 (42.5)	1355 (63.2)	1381 (54.1)	375 (63.1)	284 (89.9)	257 (78.8)	10 (66.7)
Heart diseases	322 (7.1)	123 (5.7)	465 (18.2)	97 (16.3)	‐	‐	‐
IHD	95 (2.1)	31 (1.5)	148 (5.8)	36 (6.1)	‐	6 (1.8)	0 (0)
Cardiomyopathy	54 (1.2)	21 (1.0)	203 (8.0)	38 (6.4)	9 (2.9)	‐	0 (0)
Valve disease	40 (0.9)	13 (0.6)	42 (1.7)	10 (1.7)	10 (3.2)	14 (4.3)	1 (6.7)
CHD	32 (0.7)	18 (0.8)	20 (0.8)	5 (0.8)	0 (0)	3 (0.9)	‐

Abbreviarions: CHD, congenital heart disease; IHD, ischemic heart disease.

^a^ Total targeted arrhythmias were 71 102 from 55 525 procedures. Of 40 398 AF patients, 96 could not be categorized as paroxysmal atrial fibrillation (PAF) or non‐PAF due to missing data. Of 9492 AFL patients, 43 could not be categorized as common AFL, Uncommon AFL/AT, or focal AT due to missing data.

### 3.2 Acute outcome of the ablation procedure

Table 2 shows the acute ablation outcome of all target arrhythmias. Over a 90% acute success rate was observed for all target arrhythmias except VT due to structural heart disease, and notably over a 99% success rate was achieved with pulmonary vein isolation of AF (complete success in 99.3% during the first session), IVC‐TV isthmus block for isthmus‐dependent AFL (complete success in 99.3%), and accessory pathway ablation of AVRT (complete success in 96.0%). Balloon ablation for a first pulmonary vein isolation was applied in 11 211 cases (34.7%: cryoballoon in 30.9%, hotballoon in 2.9%, and laser balloon in 0.9% of all first sessions). Over a 95% success rate was observed for slow pathway ablation of AVNRT (complete and partial success was achieved in 96.9% and 2.0%, respectively) and accessory pathway ablation of AVRT (complete success in 96.0%). A high success rate was observed for uncommon AFL/macro reentrant AT (complete and partial success was achieved in 82.5% and 11.3%, respectively), focal AT (complete and partial success was achieved in 82.2% and 12.6%, respectively), PVCs (complete and partial success was achieved in 73.9% and 18.2%, respectively), and idiopathic VT (complete and partial success was achieved in 77.6% and 15.2%, respectively). Compared to the other arrhythmias, the successful ablation rate of VT due to structural heart disease had a low but acceptable result: VT due to ischemic cardiomyopathy (complete and partial success was achieved in 50.3% and 36.2%, respectively), VT due to nonischemic cardiomyopathy (complete and partial success was achieved in 96.9% and 2.0%, respectively), and VT due to congenital heart disease (complete and partial success was achieved in 46.7% and 46.7%, respectively). We also analyzed the influence of structural heart disease on the success rate of other arrhythmias. Table 3 shows that the final success rate was similar for AF, AFL, unusual AFL/AT, AVNRT, and AVRT ablation, but the success rate for PVCs and idiopathic VT associated with structural heart disease was low (59.1% vs 77.2%, 67.0% vs 79.7%, respectively).

**TABLE 2:** Acute outcomes

	n (%)
Pulmonary vein isolation of atrial fibrillation^a^ (n = 39 480)	
Patients with a first session	32 350
Ablation system	
RF alone	21 041
Balloon alone (Cryo, hot, laser)	7201
RF + Balloon combination	4010
Success	32 133 (99.3)
Unsuccess	146 (0.5)
Already isolated	38 (0.1)
Unknown	33 (0.1)
Additional ablation	18 606 (57.5)
Patients with a second session	5771
Success	4934 (85.5)
Unsuccess	15 (0.3)
Already isolated	818 (14.2)
Unknown	4 (0.1)
Additional ablation	4398 (76.2)
Patients with a third or more session	1245
Success	785 (63.1)
Unsuccess	1 (0.1)
Already isolated	457 (36.7)
Unknown	2 (0.2)
Additional ablation	1066 (85.6)
IVC‐TV isthmus dependent atrial flutter (n = 6202)	
Success	6156 (99.3)
Unsuccess	43 (0.7)
Unknown	3 (0.1)
Uncommon atrial flutter/atrial tachycardia (n = 2063)	
Complete success	1701 (82.5)
Partial success	232 (11.3)
Unsuccess	81 (3.9)
Unknown	49 (2.4)
Focal atrial tachycardia (n = 1982)	
Complete success	1629 (82.2)
Partial success	249 (12.6)
Unsuccess	79 (4.0)
Unknown	25 (1.3)
Atrioventricular nodal reentrant tachycardia (n = 4557)^b^	
Complete success	4493 (96.9)
Partial success	94 (2.0)
Unsuccess	30 (0.6)
Unknown	21 (0.5)
Atrioventricular reentrant tachycardia (n = 2145)^c^	
Complete success	2060 (96.0)
Unsuccess	71 (3.3)
Unknown	15 (0.7)
Premature ventricular contraction (n = 2552)	
Complete success	1886 (73.9)
Partial success	465 (18.2)
Unsuccess	160 (6.3)
Unknown	41 (1.6)
Idiopathic ventricular tachycardia (n = 594)	
Complete success	461 (77.6)
Partial success	90 (15.2)
Unsuccess	31 (5.2)
Unknown	12 (2.0)
Ventricular tachycardia due to ischemic cardiomyopathy (n = 316)	
Complete success	198 (62.7)
Partial success	96 (30.4)
Unsuccess	9 (2.9)
Unknown	13 (4.1)
Ventricular tachycardia due to nonischemic cardiomyopathy (n = 326)	
Complete success	164 (50.3)
Partial success	118 (36.2)
Unsuccess	31 (9.5)
Unknown	13 (4.0)
Ventricular tachycardia due to congenital heart diseases (n = 15)	
Complete success	7 (46.7)
Partial success	7 (46.7)
Unsuccess	0 (0.0)
Unknown	1 (6.7)

Abbreviarions: IVC, inferior vena cava; RF, radiofrequency ablation; TV, tricuspid valve.

^a^ Of 39 480 patients with atrial fibrillation, 114 were not included because the number of procedures was not available.

^b^ 4638 target arrhythmias categorized by slow‐fast, fast‐slow, and other atrioventricular nodal reentrant tachycardia were included.

^c^ Kent and other atrioventricular reentrant tachycardia were included. One patient overlapped.

**TABLE 3:** Acute outcomes stratified by with or without structural heart disease

	Patients with heart diseases			Patients without heart diseases		
	n^a^	Success	%	n^a^	Success	%
AF first session	5751	5679	98.7	26 589	26 388	99.2
IVC‐TV isthmus for AFL	1792	1760	98.2	4407	4317	98.0
Uncommon AFL/AT	941	778	82.7	1122	923	82.3
Focal AT	456	370	81.1	1526	1259	82.5
AVNRT	328	309	94.2	4307	4181	97.1
AVRT	123	115	93.5	2021	1944	96.2
PVC	465	275	59.1	2086	1610	77.2
Idiopathic VT	97	65	67.0	497	396	79.7
VT due to IHD	316	198	62.7	‐	‐	‐
VT due to non‐IHD	326	164	50.3	‐	‐	‐
VT due to CHD	15	7	46.7	‐	‐	‐

Note

Success was counted by complete success.

Abbreviarions: AF, atrial fibrillation; AFL, atrial flutter; AT, atrial tachycardia; AVNRT, atrioventricular nodal reentrant tachycardia; AVRT, atrioventricular reentrant tachycardia; CHD, congenital heart disease; IHD, ischemic heart disease; IVC, inferior vena cava; PVC, premature ventricular contraction; TV, tricuspid valve; VT, ventricular tachycardia.

^a^ 19 patients (AF in 10, IVC‐TV isthmus for AFL in 3, AVNRT in 3, AVRT in 2, and PVC in 1) had missing data to structural heart disease.

### 3.3 Acute complications during hospitalization

Complications during hospitalization were observed in 1558 patients (2.8%), including major bleeding (Bleeding Academic Research Consortium: BARC criteria ≥2) in 1.1%, cerebral or systemic embolisms in 0.2%, and death in 0.1%. Most complications occurred in AF ablation cases and the difference was statistically significant in comparison to the other ablation procedures. AV block occurred in 41 cases (0.1%) and most of them were related to slow pathway ablation (Table 4). For AF ablation, we performed another analysis of the cases stratified by the AF type and number of ablation sessions. Acute complications were more frequently observed during the first session for paroxysmal AF due to the occurrence of phrenic nerve paralysis and during the first session for non‐paroxysmal AF ablation due to gastric hypomotility (Table 5). In addition, we analyzed the acute complications in patients with structural heart disease. Table 6 shows that acute complications were more frequently observed in those with structural heart disease.

**TABLE 4:** Acute complications: AF and non‐AF ablation

	All patients (n = 55 525)	AF (n = 40 398)	non‐AF (n = 15 127)	*P* value (AF vs non‐AF)
Complications during hospitalization^a^	1558 (2.8)	1290 (3.2)	268 (1.8)	<.001
Major bleeding (BARC ≥2)	625 (1.1)	498 (1.2)	127 (0.8)	<.001
Cardiac tamponade	357 (0.6)	277 (0.7)	80 (0.5)	.040
Embolism	112 (0.2)	98 (0.2)	14 (0.09)	<.001
Phrenic nerve paralysis	249 (0.5)	240 (0.6)	9 (0.06)	<.001
Esophagus	95 (0.2)	93 (0.2)	2 (0.01)	<.001
Esophagus ulcer	31 (0.1)	31 (0.1)	0 (0)	
Gastric hypomotility	64 (0.1)	62 (0.2)	2 (0.01)	
Atrioesophageal fistula	1 (0.001)	1 (0.002)	0 (0)	
Pericarditis	87 (0.2)	75 (0.2)	12 (0.08)	.005
Sick sinus syndrome	87 (0.2)	71 (0.2)	16 (0.1)	.063
Atrioventricular block	41 (0.1)	9 (0.02)	32 (0.2)	<.001
Death during hospitalization^b^	63 (0.1)	37 (0.09)^‡^	26 (0.2)	.012
Cardiac death	31 (0.06)	17 (0.04)	14 (0.09)	
Related to ablation therapy	6 (0.01)	4 (0.01)	2 (0.013)	
Noncardiac death	20 (0.04)	11 (0.03)	9 (0.06)	
Related to ablation therapy	1 (0.002)	0 (0)	1 (0.007)	
Unknown	12 (0.02)	9 (0.02)	3 (0.02)	

Abbreviarions: AF, atrial fibrillation; BARC, Bleeding Academic Research Consortium.

^a^ Of 55 525 patients, 92 (70 in atrial fibrillation [AF] patients, 22 in non‐AF) had missing data due to complications during the hospitalization.

^b^ 101 patients (76 in AF patients; 25 in non‐AF) had missing data to death during the hospitalization.

**TABLE 5:** Acute complications stratified by AF type and the number of ablation sessions

	pAF			non‐pAF		
	First session	Second or more	*P* value	First session	Second or more	*P* value
	(n = 19 712)	(n = 4754)		(n = 12 780)	(n = 2915)	
Complications during hospitalization^a^	677 (3.4)	116 (2.4)	.001	424 (3.3)	72 (2.5)	.018
Major bleeding (BARC ≥2)	264 (1.3)	52 (1.1)	.178	154 (1.2)	28 (1.0)	.266
Cardiac tamponade	146 (0.7)	33 (0.7)	.736	81 (0.6)	17 (0.6)	.754
Embolism	44 (0.2)	5 (0.1)	.145	43 (0.3)	6 (0.2)	.356
Phrenic nerve paralysis	175 (0.9)	17 (0.4)	<.001	36 (0.3)	12 (0.4)	.264
Esophagus	43 (0.2)	7 (0.2)	.473	42 (0.3)	1 (0.03)	.003
Esophagus ulcer	18 (0.09)	3 (0.06)	.783	9 (0.07)	1 (0.03)	.700
Gastric hypomotility	25 (0.1)	4 (0.08)	.638	33 (0.3)	0 (0)	.002
Atrioesophageal fistula	1 (0.01)	0 (0)	1.000	0 (0)	0 (0)	‐
Pericarditis	41 (0.2)	6 (0.1)	.354	25 (0.2)	3 (0.1)	.463
Sick sinus syndrome	21 (0.1)	13 (0.3)	.014	28 (0.2)	9 (0.3)	.395
Atrioventricular block	5 (0.03)	2 (0.04)	.628	2 (0.02)	0 (0)	1.000
Death during hospitalization^b^	18 (0.09)	3 (0.06)	.783	14 (0.11)	2 (0.07)	.752
Cardiac death	8 (0.04)	2 (0.04)	1.000	7 (0.05)	0 (0)	.362
Noncardiac death	6 (0.03)	0 (0)	.604	4 (0.03)	1 (0.03)	1.000
Unknown	4 (0.02)	1 (0.02)	1.000	3 (0.02)	1 (0.03)	.560

Abbreviarions: BARC, Bleeding Academic Research Consortium; pAF, paroxysmal atrial fibrillation.

^a^ 117 (53 in paroxysmal AF [pAF], 64 in non‐PAF) could not categorized as first or ≥second session due to missing data. About 24 patients (18 in pAF patients; 6 in non‐pAF) had missing data to complications during hospitalization.

^b^ 3 patients with pAF and 4 patients with non‐pAF had missing data to death during hospitalization.

**TABLE 6:** Acute complications stratified by patients with or without structural heart disease

	Patients with heart diseases (n = 10 562)^a^	Patients without heart diseases (n = 44 851)^a^	*P* value (with vs without)
Complications during hospitalization^a^	426 (4.0)	1132 (2.5)	<.001
Major bleeding (BARC ≥2)	159 (1.5)	466 (1.0)	<.001
Cardiac tamponade	93 (0.9)	264 (0.6)	.001
Embolism	27 (0.3)	85 (0.2)	.173
Phrenic nerve paralysis	54 (0.5)	195 (0.4)	.290
Esophagus	20 (0.2)	75 (0.2)	.621
Esophagus ulcer	5 (0.05)	26 (0.06)	.821
Gastric hypomotility	15 (0.1)	49 (0.1)	.372
Atrioesophageal fistula	0 (0)	1 (0.002)	1.000
Pericarditis	15 (0.1)	72 (0.2)	.666
Sick sinus syndrome	28 (0.3)	59 (0.1)	.002
Atrioventricular block	17 (0.2)	24 (0.05)	.001
Death during hospitalization^b^	34 (0.3)	29 (0.06)	<.001
Cardiac death	25 (0.2)	6 (0.01)	<.001
Related to ablation therapy	3 (0.03)	3 (0.01)	.088
Noncardiac death	7 (0.07)	13 (0.02)	.084
Related to ablation therapy	0 (0)	1 (0.002)	1.000
Unknown	2 (0.02)	10 (0.02)	1.000

Abbreviarion: BARC, Bleeding Academic Research Consortium.

^a^ Of 55 413 patients, 70 patients (15 patients with heart diseases; 55 patients without heart diseases) had missing data to complications during hospitalization.

^b^ 12 patients (2 patients with heart diseases; 10 patients without heart diseases) had missing data to death during hospitalization.

## 4 DISCUSSION

To the best of our knowledge, this nationwide annual registry was the largest (55 525 procedures per year) to have collected the efficacy and safety points of all ablation procedures. The major findings of the present study were as follows. (a) Two‐thirds of all catheter ablation targets were AF. (b) Balloon ablation was used in 34.7% of the first procedures for PV isolation of AF. (c) Over a 90% acute success was observed among all arrhythmias except for VT due to structural heart disease. (d) Acute complications during hospitalization occurred in 2.8%, including major bleeding in 1.1%, embolisms in 0.2%, and all cause death in 0.1%. The complication rate significantly differed between AF ablation and other ablation procedures.

During the past two decades, the number of ablation cases has dramatically increased. Data from a Japanese nationwide database using the administrative case‐mix Diagnostic Procedure Combination (DPC) system (ie, the Japanese Registry Of All cardiac and vascular Diseases [JROAD]‐DPC) has reported that the total number of ablation cases has exceeded 70 000 and is still increasing.^3^ However, the details of the procedures (type of targeted arrhythmias, success rate or complications during the ablation procedure, and current strategy, etc) have been lacking. Accordingly, the J‐AB registry was conducted to clarify the efficacy and safety in the real‐world setting.^4^ This study was started in August 2017 and was expected to initially collect data from around 250 hospitals, but the number of participating medical institutions increased to over 350 hospitals by the end of 2018.

### 4.1 Comparison with other studies

Similar large‐scale ablation registries have been reported in other countries. In Spain, the Spanish Catheter Ablation Registry was started in 2000 and reported the annual features of catheter ablation every year.^5^ That registry consisted of 16 566 procedures per year in 2018, but they used questionnaires to collect the data from 100 selected centers. In Germany, the German Ablation Quality Registry was conducted to clarify the specific ablation procedures for selected arrhythmias (AF,^6^ PVC,^7^ and PSVT^1^) but that registry also used the data from 52 selected hospitals. In the United States, large‐scale ablation data were reported (50 969 ablation recipients during the years of 2011 to 2014), but that report used the retrospective data from the Nationwide Inpatient Sample (NIP) database, and specifically collected in‐hospital complications associated with AF ablation.^8^ In Japan, we previously reported similar registry results focused on AF ablation, named the Japanese Catheter Ablation Registry of Atrial Fibrillation (J‐CARAF^9^: total 10 795 AF ablation session data from a mean of 165 hospitals), but the data were still obtained from selected hospitals and focused on the complications during AF ablation. Accordingly, the current J‐AB registry was quite unique in that it collected data on all ablation cases from 369 participating hospitals to examine the efficacy and safety.

### 4.2 Acute successful rate of ablation procedures

In this J‐AB registry, two‐thirds of the ablation targets were AF and that number was quite a high prevalence as compared to the other countries.^1^, ^2^, ^6^ The success rate of the target arrhythmias was more than 90% for atrial arrhythmias (AF, AFL, AT, and PSVT) but was below 90% for ventricular arrhythmias, especially in patients with ischemic or nonischemic heart diseases. This trend was similar to the other countries.^5^, ^6^ However, the success rate for atrial arrhythmias (AF, AFL, AT, and PSVT) did not differ between the patients with and without structural heart disease. Balloon ablation for pulmonary vein isolation became available since 2014, and 34.7% of the first AF ablation procedures used balloons in 2018. Balloon ablation will become more popular in Japan.

### 4.3 Acute complication rate of the ablation procedures

The most important results were the rate of acute complications during the procedures. In this registry, acute complications were observed in 2.8% of all procedures. Major bleeding was 1.1%, cardiac tamponade 0.6%, embolic complications 0.2%, esophageal injury 0.2%, pericarditis 0.2%, sick sinus syndrome 0.2%, and AV block 0.1%. In particular, complications other than AV block were more commonly observed in AF ablation, and acute complications occurred in 3.2%, including major bleeding in 1.2%, cardiac tamponade in 0.7%, embolisms in 0.2%, phrenic nerve injury in 0.6%, and esophageal injury in 0.2%. Compared to the previous reports in other countries, acute complications during hospitalization were similar or low. In the Spanish Catheter Ablation Registry, the rate of all complications was 2.0%‐2.6% for all ablation procedures, and 3.4%‐5.1% for AF ablation.^5^ In the German Ablation Registry, minor and nonfatal complications occurred in 3.5% (≥75 years of age) and 4.2% (<75 years of age) of AF ablation cases, respectively.^6^ In the US report, the overall complication rate was 5.46% and the in‐hospital mortality rate was 0.15% for AF ablation.^8^ In the J‐CARAF during the years from 2011 to 2016, total major complications occurred in 3.0% of the AF ablation procedures.^9^ We need to pay more careful attention to avoid periprocedural complications during AF ablation. Among the acute complications during AF ablation, in this report, we performed another analysis of cases stratified by the AF type and number of ablation sessions. More acute complications occurred during the first session as compared to the second or greater sessions. Among those, phrenic nerve paralysis occurred more frequently during paroxysmal AF ablation, and gastric hypomotility occurred more frequently during non‐paroxysmal AF ablation (Table 5). We speculated that phrenic nerve paralysis (n = 211) was due to balloon ablation (158 of 211 case, *P* < .001; data not shown), but could not find any factors associated with the gastric hypomotility in this study. Further detailed data would be needed to explain that. In particular, the death rate during VT ablation associated with structural heart disease (non‐idiopathic VT ablation) was more frequently observed in 12 of 34 cases, as compared to idiopathic VT ablation (1 of 29 cases, *P* = .002; data not shown). Structural heart disease is one of the most important factors that raises the complication/death rate after catheter ablation.

### 4.4 Limitations

There were several important limitations to this registry. First, this registry was voluntary, and more than 70 000 ablation cases were reported from the JROAD‐DPC data, thus, this registry did not cover all ablation procedures in Japan. Second, esophageal injury and pulmonary vein stenosis usually occurred late after the ablation procedure, thus, the number stated in this study would have been underestimated in this registry. Third, data monitoring was not performed in this report, thus, the true numbers might differ from this study. Careful attention would be needed to interpret the present results that might have been biased by the limitations inherent to observational studies.

## 5 CONCLUSION

The J‐AB registry 2018 for the first time provided the real‐world data regarding the outcome and acute complications of catheter ablation of the various types of arrhythmias in Japan. Ablation therapy is a safe and effective treatment for cardiac arrhythmias, but you still need to pay attention to the development of serious complications during AF procedures.

## CONFLICT OF INTEREST

Kengo Kusano: Speaker honoraria from DAIICHI SANKYO COMPANY, Ltd., Japan, Bristol‐Myers Squibb, Biotronik Japan, and Medtronic Japan, and research grants from Medtronic Japan and EP‐CRSU Co., Ltd. Teiichi Yamane: Speaker honoraria from DAIICHI SANKYO COMPANY, Ltd., Japan, Boehringer Ingelheim, Abbott Japan, Bristol‐Myers Squibb, Medtronic Japan, and Japan LifeLine, and research grants from Boehringer Ingelheim. Koichi Inoue: Speaker honoraria from DAIICHI SANKYO COMPANY, Ltd., Japan, Bristol‐Myers Squibb, Bayer Yakuhin, Nihon Boehringer Ingelheim, Johnson and Johnson KK, and Medtronic Japan. Morio Shoda: Speaker honorarium from Medtronic Japan, and financial endowments to our clinical research division from Biotronik Japan, Medtronic Japan, Boston Scientific Japan, and Abbott Japan. Akihiko Nogami: Speaker honoraria from Abbott, Biosense Webster, and Daiichi‐Sankyo; an endowment from Medtronic and DVX. None: MT, YMN, YM. IRB approval number: M28‐114‐7, IRB approved date at Dec 21, 2016 at the National Cerebral and Cardiovascular Center Japan.
